# Evaluation of Surgical Site Infection After Elective Surgeries at a Tertiary Care Hospital

**DOI:** 10.7759/cureus.50844

**Published:** 2023-12-20

**Authors:** Rajen Rajak, Nishith S Mandal

**Affiliations:** 1 General Surgery, Vardhman Mahavir Medical College and Safdarjung Hospital, New Delhi, IND; 2 Surgery, Vardhman Mahavir Medical College and Safdarjung Hospital, New Delhi, IND

**Keywords:** wound category, southampton score, postoperative wound infection, elective surgical procedures, surgical site infections (ssi)

## Abstract

Surgical site infections (SSI) are commonly seen in surgical practice and are the main cause for concern in post-operative patients. There are many risk factors that predispose to the development of SSI. However, the occurrence of SSI in patients undergoing elective class I and class II surgeries, which are considered clean surgeries with minimal contamination, is an important issue bothering the surgeons. SSI are also responsible for increased morbidity due to wound dehiscence, thus prolonging hospital stays and often leading to inconvenience to patients. We hereby present a study to highlight and address this important issue of SSI in our institute. All patients above 12 years of age who underwent elective class I and class II surgeries in the department of general surgery were included in this observational study. After surgery, local examination of the incision or wound site and grading of the SSI were done using the Southampton Wound Grading System (SWGS). Our results showed that 90% of the patients had normal healing, according to SWGS. We found that the incidence of SSI was lower in patients who underwent alternate-day dressing of their wound as compared to daily dressing. Another interesting finding was that the incidence of SSI was lower in patients in whom wound dressing was done with transparent film dressing as compared to povidone-iodine-guaze dressing. We concluded our study by finding that the incidence of SSI after elective class I and class II surgeries in our hospital was quite low, at 10%.

## Introduction

SSI is one of the most common hospital infections and contributes substantially to post-operative morbidity and mortality. SSI is defined by the Center for Disease Control and Prevention (CDC) as a wound infection that occurs within 30 days of an operative procedure or within a year if an implant is left in place and the infection is thought to be secondary to surgery [1]. Wound infections, seromas, and wound dehiscence are the most common complications in post-operative wounds [2]. SSI are responsible for wound dehiscence leading to a burst abdomen, which ultimately leads to poor cosmetic scars, re-admissions, and increased rates of secondary suturing and incisional hernias later [3].

According to the CDC guidelines, surgical wounds are classified into four types: 1) Class I/Clean wound. 2) Class II/Clean-Contaminated Wound 3) Class III/Contaminated wound. 4) Class IV/Dirty-Infected Wound [1]. The main purpose of our study was to find out the incidence of SSI in the first two classes of surgical wounds. The significance of this study arises from the fact that these elective class I and II surgeries are relatively clean procedures with minimal contamination, and hence, SSI should be less after such surgeries. We hypothesized that the occurrence of SSI following elective class I and II surgeries at a tertiary care center was low.

As per the study by Kumar A et al., SSI was prevalent in 12.5% of patients, and among the 3 types, superficial incision SSI was most prevalent, followed by deep incisional SSI, and finally, organ/space SSI [4]. SSI rates have been reported to be <2% for clean (class I), 5%-15% for clean-contaminated (class II), 15%-30% for contaminated (class III), and >30% for dirty infected wounds (class IV) [5]. Various scoring systems have been developed to objectively classify SSI. ASEPSIS (Additional treatment, serous discharge, erythema, purulent exudate, separation of deep tissues, isolation of bacteria, and staying inpatient for prolonged periods) and SWGS are commonly used. SWGS was originally designed by Bailey et al. in 1992 to assess hernia wounds [6]. The aim of our study was to evaluate the incidence of SSI using SWGS in class I and class II elective surgeries [7].

## Materials and methods

The study was conducted in the departments of general surgery at Vardhman Mahavir Medical College and Safdarjung Hospital, New Delhi. The study design was a prospective observational-cohort study. The study period was from March' 2021 to March' 2023. All patients above 12 years of age undergoing elective surgeries in Class I and Class II (clean and clean-contaminated) categories were included in the study. Patients undergoing surgery for trauma were excluded from the study.

The methodology adopted was that the patient's were assessed pre-operatively, intra-operatively, and post-operatively. The pre-operative evaluation of the patients included a detailed history and a thorough clinical examination. Details of the procedure were done, and any intra-operative, anesthetic, or surgical complications were recorded. Post-operatively, the vital signs of the patient were monitored daily during the morning rounds by the resident surgeon on duty. A local examination of the incision site was also done daily, and any discharge or soakage of dressing material and signs of inflammation, e.g., wound edema or redness, raised local temperature, tenderness, induration, etc., were noted, and the grading of the SSI was done accordingly using the SWGS. The relevant laboratory tests, as indicated during local examination (total leukocyte count, differential leukocyte count, culture/sensitivity of wound discharge, etc.), and radiological investigation (ultrasonography to find out deep or organ abscess cavities whenever indicated) were also done to diagnose and start appropriate treatment. Patients were followed up weekly for 30 days after discharge after an elective surgical procedure and monthly for up to one year if any implant was placed for the development of SSI.

SWGS is mentioned in Table 1. 

**Table 1:** Southampton wound grading system (SWGS)

	Grade	Appearance
0	Normal healing	
I	Normal healing with mild bruising or erythema	A – some bruising
		B – considerable bruising
		C – mild erythema
II	Erythema plus other signs of inflammation	A – at one point
		B – around sutures
		C – along wound
		D – around wound
III	Clear or haemoserous discharge	A – at one point only (< 2 cm)
		B – along wound (> 2 cm)
		C – large volume
		D – prolonged (> 3 cm)
IV	Pus/purulent discharge	A – at one point only (< 2 cm)
		B – along wound (> 2 cm)
V	Deep or severe wound infection with or without tissue breakdown;	

For calculating the sample size, the study by Patel SM et al. was taken as a reference study, in which out of 200 patients, 32 patients developed SSI (16%). Out of those 32 infected cases, 28 were culture-positive (87.5%), while four were culture-negative (12.5%) [8]. Taking this value as a reference, the minimum required sample size with a 7.5% margin of error and a 5% level of significance was 92 patients. To reduce the margin of error, the total sample size was rounded off to 100 patients. The formula used was: N = (i(1 -i))/(ME/Zα)2 , where "Zα" is the value of Z at a two-sided alpha error of 5%, "ME" is the margin of error, and "i" is the incidence rate of SSI. So N = ((.16*(1-0.16))/(0.075/1.96)2= 91.79= 92 patients (approx).

For statistical analysis, categorical variables were presented in numbers and percentages (%), and continuous variables were presented as mean ± standard deviation (SD) and median. The normality of the data was tested by the Kolmogorov-Smirnov test. If normality was rejected, then a non-parametric test was used. In statistical tests, quantitative variables were compared using the unpaired t-test or Mann-Whitney test (when the data sets were not normally distributed). Qualitative variables were compared using the chi-square test or Fisher's exact test. Univariate logistic regression was used to find out the risk factors for SSI. A p value of <0.05 was considered statistically significant. The data was entered in an MS Excel spreadsheet, and analysis was done using IBM Corp. Released 2012. IBM SPSS Statistics for Windows, Version 21.0. Armonk, NY: IBM Corp.

## Results

The age group of the patients ranged from 14 to 82 years, with a mean (±SD) of 39.78 (±14.88) years and a median (IQR) of 41 years. 33% of the patients were <30 years old, 41% were between 30 and 50 years old, and 26% were >50 years old. Among 100 patients, 76% were females and 24% were males. The duration of hospital stay ranged from 2-27 days, with a mean (±SD) of 5.53 (±3.48) days and a median (IQR) of four days. The duration of hospital stay was <7 days for 78% of the patients and >7 days for 22% of the patients. With reference to co-morbidities, around 14% of the patients had hypertension, 11% had diabetes mellitus, 4% had tuberculosis, 3% had hypothyroidism, and 1% each had hyperthyroidism and asthma. The serum albumin level in the patients ranged from 2.7 to 4.5 g/dl, with a mean of 3.62 (± 0.37) g/dl and a median (IQR) of 3.6 (3.4, 3.9) g/dl. 33% of the patients had hypoalbuminemia (serum albumin <3.5 g/dl), while 67% of the patients had normal albumin levels (3.5-5.5 g/dl).

**Figure 1:**
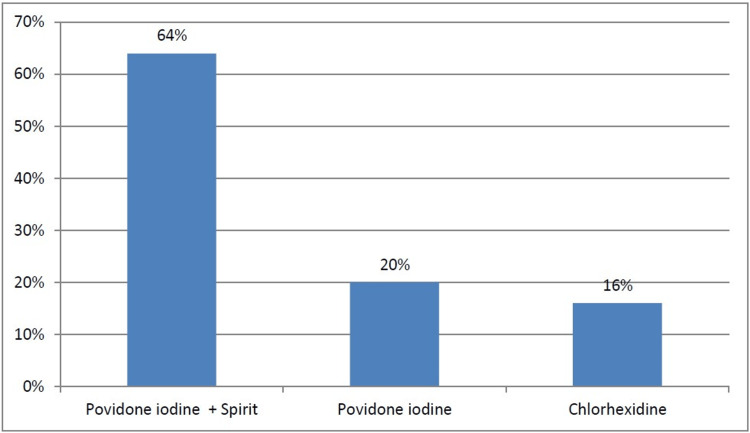
Skin preparation for patients

The skin preparation was done with povidone iodine+spirit in 64% of the patients, povidone iodine alone in 20% of the patients and with chlorhexidine in 16% of the patients, as shown in Figure 1. 

**Figure 2:**
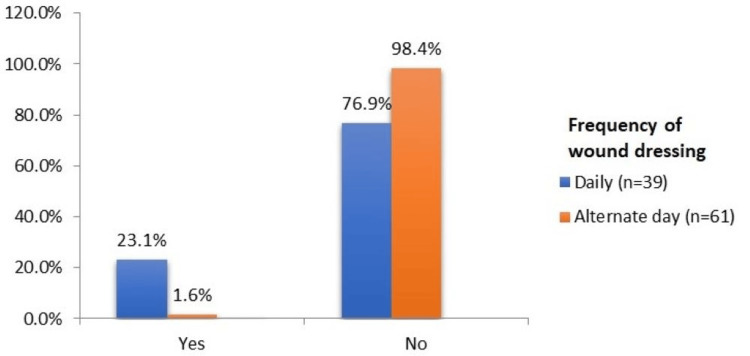
SSI according to the frequency of dressing

The frequency of wound dressing was daily in 39% of the patients, and alternate-day dressing was done in 61% of the patients, as shown in Figure 2. However, the incidence of SSI was higher in patients undergoing daily dressing (23.1%) as compared to those with alternate day dressing (1.6%). 

**Figure 3:**
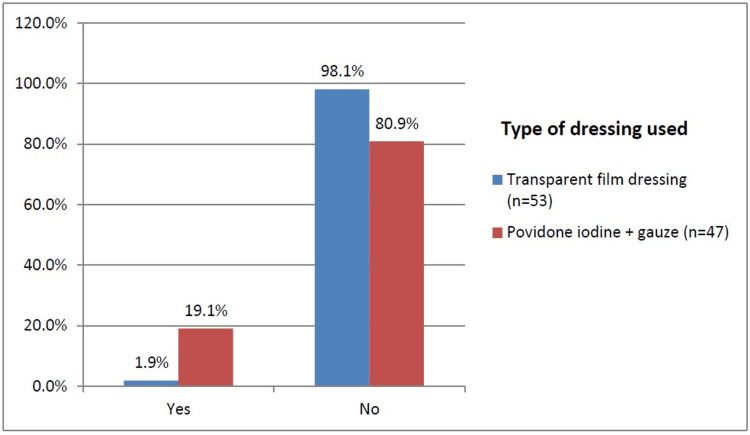
SSI according to the type of dressing used

Transparent film dressing was used as a dressing material in 53% of the patients, whereas povidone iodine+gauze was used as dressing for 47% of the patients, as shown in Figure 3. The incidence of SSI was higher in povidone iodine+guaze dressing (19.1%) than in the transparent film dressing patients (1.9%).

The overall incidence of SSI was 10%. According to SWGS, 90% of the patients were graded 0, i.e., they had normal healing. While 1% each were graded IA (they had some bruising), grade IB (they had considerable bruising), grade IC (they had mild erythema), grade IIB (they had erythema plus other signs of inflammation around sutures), grade IIIA (they had clear/haemoserous discharge at one point only <2 cm), grade IIIB (they had clear/haemoserous discharge along wound >2 cm), and grade IIID (they had clear/haemoserous discharge for prolonged period >3 days). 2% of patients were graded IVB (they had pus/ purulent discharge along the wound >2 cm), and 1% were graded V (they had a deep/severe wound infection with or without tissue breakdown). The grading of SSI by SWGS is shown in Table 2. 

**Table 2:** Southampton grades of SSI

Southampton score	Frequency	Percentage
0	90	90.0%
IA	1	1.0%
IB	1	1.0%
IC	1	1.0%
IIB	1	1.0%
IIIA	1	1.0%
IIIB	1	1.0%
IIID	1	1.0%
IVB	2	2.0%
V	1	1.0%

**Figure 4:**
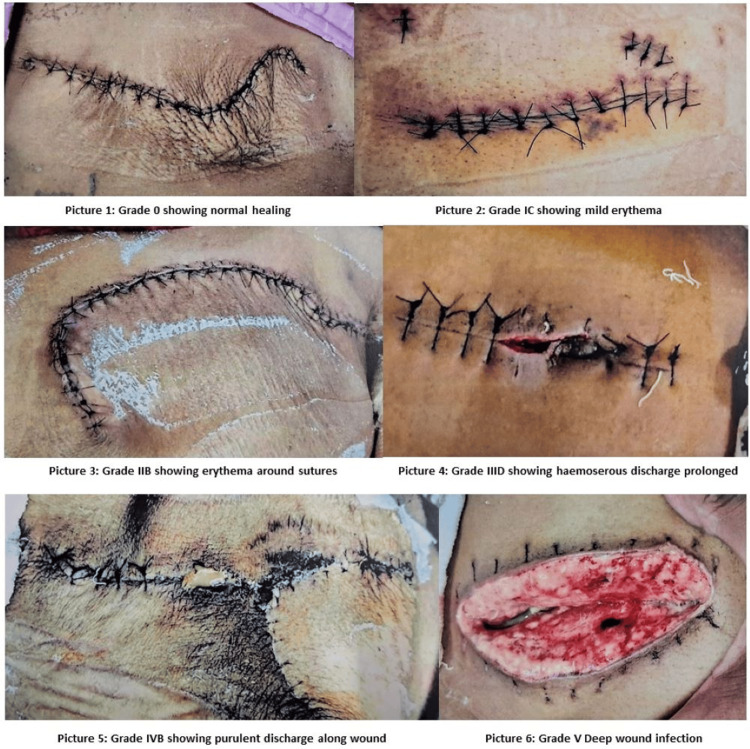
Southampton grades of SSI Picture 1: Patient of modified radical mastectomy showing normal wound healing, corresponding to Southampton SSI grade "0". Picture 2: Patient of hepatic hydatid cystectomy showing mild erythema along the lower part of the incision after removal of cystic and pelvic drains, corresponding to Southampton SSI grade "I C". Picture 3: Patient with wide local excision of soft tissue sarcoma over the right scapular region showing erythema along vertical sutures, corresponding to Southampton SSI grade "II B". Picture 4: Patient with umbilical mesh hernioplasty showing prolonged hemo-serous discharge necessitating removal of a suture above the umbilicus, corresponding to Southampton SSI grade "III D". Picture 5: Patient of excision of scalp dermoid showing purulent discharge along the incision site for more than 2 cm, corresponding to Southampton SSI grade "IV B". Picture 6: Patient of laparotomy in a case of illeal resection anastomosis showing burst abdomen with rectus sheath dehiscence and congested bowel loop with slough, corresponding to Southampton SSI grade "V". "All Images credit: Rajen Rajak".

Among the 10 patients who developed SSI, seven patients had superficial incisional infection, and three patients had deep incisional infection. Few of the Southampton grades of SSI seen in patients are depicted in the following pictures in Figure 4.

**Figure 5:**
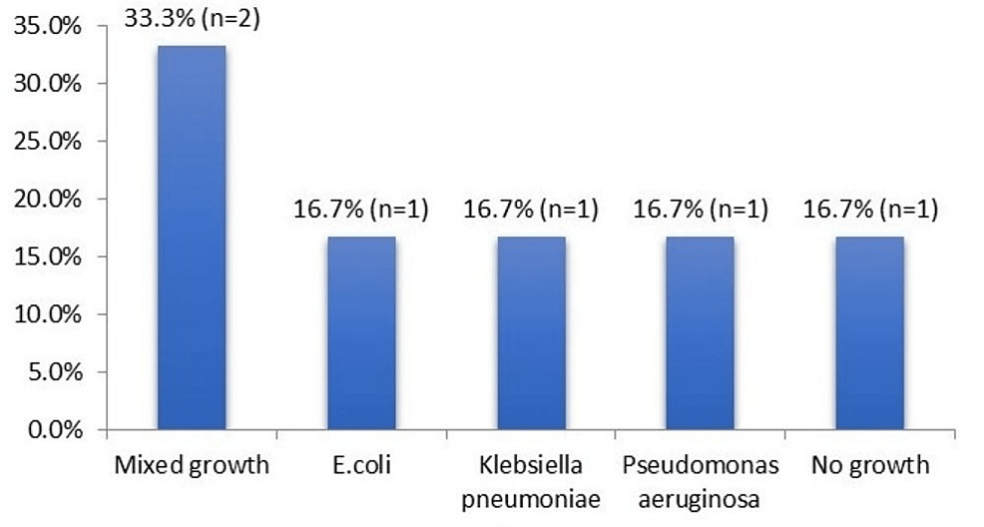
Culture and sensitivity patterns in SSI

The culture and sensitivity pattern among six patients with SSI who underwent culture showed mixed growth in the cultures of two patients, while *E. coli*, *Klebsiella pneumonia*, and *Pseudomonas aeruginosa* were isolated in one patient each, and no growth was seen in one patient, as shown in Figure 5.

The following Table 3 summarizes the incidence of SSI seen in different conditions. 

**Table 3:** SSI in different conditions

Variables		Surgical site infection		P-value
		Yes	No	
Mean ± SD age (years)		44.20±17.86	39.29±14.55	0.325
Age group	<=30 years (n=33)	3(9.1%)	30(90.9%)	0.158
	31 to 50 years (n=41)	2(4.9%)	39(95.1%)	
	>50 years (n=26)	5(19.2%)	21(80.8%)	
Sex	Female (n=76)	9(11.8%)	67(88.2%)	0.482
	Male (n=24)	1(4.2%)	23(95.8%)	
Median (IQR) duration of hospital stay		5.5(4,7)	4(4,5)	0.254
Duration of hospital stay	<7 days (n=78)	6(7.7%)	72(92.3%)	0.296
	>=7 days (n=22)	4(18.2%)	18(81.8%)	
Any co-morbidities	Present (n=32)	3(9.4%)	29(90.6%)	1.000
	Absent (n=68)	7(10.3%)	61(89.7%)	
Skin Preparation	Povidone iodine+Spirit (n=64)	6(9.4%)	58(90.6%)	0.113
	Povidone iodine (n=20)	4(20%)	16(80%)	
	Chlorhexidine (n=16)	0(0%)	16(100%)	
Frequency of Wound Dressing	Daily (n=39)	9(23.1%)	30(76.9%)	0.002
	Alternate day (n=61)	1(1.6%)	60(98.4%)	
Type of dressing used	Transparent film dressing (n=53)	1(1.9%)	52(98.1%)	0.011
	Povidone iodine+gauze (n=47)	9(19.1%)	38(80.9%)	
Mean ± SD serum albumin (g/dl)		3.70±0.53	3.61±0.35	0.615
Serum albumin	<3.5 g/dl (n=33)	3(9.1%)	30(90.9%)	1.000
	3.5 to 5.5 g/dl (n=67)	7(10.4%)	60(89.6%)	

In the univariate analysis shown in Table 4, frequency of wound dressing and type of dressing used were found to be statistically significant predictors of SSI (p<0.05). Patients with daily wound dressing were 14.08 times (crude RR 14.08, 95% CI 1.78-111.11) more likely to have SSI as compared to patients with alternate day wound dressing. Patients with povidone iodine+gauze dressing were 10.15 times (crude RR 10.15, 95% CI 1.29-80.10) more likely to have SSI as compared to patients with transparent film dressing. 

**Table 4:** Univariate logistic regression to assess predictors of SSI

Variables	Univariate model	
	Crude RR (95% CI)	P-value
Age (years)	1.02(0.98-1.06)	0.347
Gender: Male	0.35(0.04-2.78)	0.322
Any co-morbidities: Present	0.91(0.24-3.52)	0.892
Duration of hospital stay (in days)	1.07(0.95-1.2)	0.258
Skin Preparation: Povidone iodine+Spirit	0.47(0.13-1.66)	0.240
Skin Preparation: Chlorhexidine	0(0-Inf)	0.994
Frequency of wound dressing: Daily	14.08(1.78-111.11)	0.012
Type of dressing used: Povidone iodine+gauze	10.15(1.29-80.10)	0.028
Serum albumin (g/dl)	1.80(0.33-9.71)	0.493

## Discussion

SSI is a major concern after any surgery. SSI typically occurs within 30 days after surgery. As per CDC guidelines, surgical wounds are classified into four types: 1) Class I/Clean wound. 2) Class II/Clean-Contaminated wound. 3) Class III/Contaminated wound. 4) Class IV/Dirty-Infected wound [1]. The CDC describes three types of SSI: 1) Superficial incisional SSI: This infection occurs just in the area of the skin where the incision was made. 2) Deep incisional SSI: This infection occurs beneath the incision area in muscle and the tissues surrounding the muscles. 3) Organ or space SSI: This type of infection can be in any area of the body other than the skin, muscles, and surrounding tissue that was involved in the surgery. This includes a body organ or a space between organs. Elective class I and class II surgeries are considered to be relatively clean surgeries. Hence, the occurrence of SSI in these patients is a real worry for any surgeon. In our study, elective class I and class II surgical interventions, in decreasing order, were cholecystectomy, mastectomy, hernioplasty, thyroidectomy, appendicectomy, resection-anastomosis of the bowel, etc. The present study was carried out to evaluate the incidence and different factors responsible for the occurrence of SSI following elective class I and II surgeries.

Age

Most patients (41%) were found in the age group of 31-50 years, followed by 33% in the age group of <31 years, followed by 26% in the age group >50 years, with a mean age of 39.78 (±14.38) years and a p-value of 0.325, which was statistically significant. 9.1% of 33 patients in the age group <31 years, 4.9% of 41 patients within the age group 31-50 years, and 19.2% of 26 patients with an age >50 years had SSI. There was a statistically significant difference in the proportion of SSI according to age groups (p>0.05). In a similar study by Kaye KS et al., the effect of increasing age on the risk of developing SSI revealed a significant relationship. Increasing age independently predicted an increased risk of SSI until the age of 65 years. After the age of ⩾65 years, increasing age independently predicted a decreased risk of SSI (p = 0.006) [9].

Gender

Most of the patients (76%) were females, and 24% were males. In the study, 11.8% of the female patients and 4.2% of the male patients developed SSI. However, the difference in the incidence of SSI among male and female patients was not statistically significant, with a p-value of 0.482. The higher incidence of SSI in females is due to the fact that the most commonly performed surgery was laparoscopic cholecystectomy, and females are more affected by gallstone disease. Our finding was similar to the study conducted by Mukagendaneza MJ et al., where the incidence of SSI was 13.3% among male patients compared to 7.7% among females. The difference in the incidence of SSI among male and female patients in their study was also not statistically significant, with a p-value of 0.165 [10].

Duration of hospital stay

The duration of hospital stays ranged from 2-27 days, with a mean duration of 5.53 (±3.48) days. 78% of patients had a hospital stay of <7 days, while 22% had >7 days of hospital stay. Only 7.7% of the patients with hospital stays of <7 days developed SSI, while a higher number of 18.2% of patients with hospital stays of >7 days developed SSI, which was statistically significant with a p-value of 0.296. Kowli et al. also found similar findings of SSI being lower at 17.4% when the hospital stay was 0-7 days and a higher incidence of SSI of 71.4% when the stay was >21 days [11]. Berríos-Torres SI et al. demonstrated that an increase in hospital stay predisposed an individual to a 1.76% risk of acquiring SSI. With an increase in the duration of pre- and post-operative hospital stays, the risk of SSI increases proportionately [1].

Skin preparation

Pre-operative skin preparation was done in 64% of patients with povidone iodine+spirit, followed by povidone iodine alone in 20% of patients and chlorhexidine in 16% of patients, as per availability in the hospital. Around 9.4% of patients with povidone iodine+spirit skin preparation, 20% of patients with only povidone iodine skin preparation, and none of the patients with chlorhexidine skin preparation developed SSI, which was statistically significant with a p-value of 0.113. The overall rate of SSI was significantly lower in the chlorhexidine group than in the povidone-iodine group. In a similar study conducted by Darouiche RO et al., the overall rate of SSI was significantly lower in the chlorhexidine group than in the povidone iodine group (9.5% vs. 16.1%) [12]. Chlorhexidine was significantly more protective than povidone iodine against both superficial incisional SSI (4.2% vs. 8.6%) and deep incisional SSI (1% vs. 3%), but not against organ-space infections (4.4% vs. 4.5%). 

Frequency of wound dressing

39% of patients underwent daily dressing, while 61% of patients had alternate day dressing, depending on the type of surgery. More than 23.1% of patients with daily dressing and only 1.6% of patients with alternate day dressing developed SSI. There was a significant difference in the proportion of SSI according to the frequency of wound dressing, with a p-value of 0.002, as exposure of the incision site to the environment while dressing was comparatively more common in daily dressing as compared to patients requiring alternate day dressing.

Type of dressing material

53% of patients had transparent film dressing, while 47% had dressing done with povidone, iodine, and gauze. But, only 1.9% of patients with transparent film dressing developed SSI, while more than 19.1% of patients with povidone iodine+gauze dressing had SSI. There is a significant difference in the proportion of SSI according to the type of dressing material used, with a p-value of 0.011, as transparent film dressing is seen to offer better occlusive barrier protection than povidone-iodine-guaze dressing.

Culture and sensitivity pattern

Among the 10 patients who developed SSI, culture and sensitivity reports in 60% of patients revealed mixed growth in 33.3% of cases, E. coli, K. pneumoniae, and P. aeruginosa, and no growth in the remaining cases (16.7% each). The study shows that most SSIs are caused by a combination of microorganisms rather than a single pathogen, and hence, appropriate broad-spectrum antibiotics should be started if indicated, pending a C/S report. Our findings were similar to those of the study conducted by Patel S et al., in which, out of 200 patients, 32 developed SSI, and out of these, 28 were culture-positive, while the remaining four were culture-negative [8].

Study limitations

This was a single-center study, which limits its generalizability to different healthcare systems. Additionally, the study was undertaken in the department of general surgery, so it may not correctly reflect the occurrence of SSI in other surgical departments with different patient profiles. The absence of comparative studies between different groups limits its ability to predict the superiority of one treatment modality over another in the prevention of SSI.

## Conclusions

SSIs are commonly seen in surgical practice and are the main cause of post-operative wound complications. The occurrence of SSI, even after relatively clean elective class I and class II surgeries, remains a major concern for surgeons. We conclude that the incidence of SSI in elective class I and class II surgeries at our tertiary care center is low, at around 10%. SSIs are persistent and preventable health-care-associated infections. Based on our study, we can recommend that pre-operative skin painting be done with an alcohol-containing antiseptic like chlorhexidine rather than povidone iodine. Transparent film dressing should be used for better occlusive dressing of the wounds rather than povidone, iodine, and gauze. The frequency of dressing for clean wounds should be on alternate days rather than on a daily basis to reduce the incidence of SSI. 

A few other general measures that can be followed in addition to those above for the reduction of SSI are highlighted. Patients should bathe with soap or an antiseptic agent the night before surgery. Nursing staff should remove patients' hair with a clipper or depilatory agent outside the operating room. Before the surgical incision, patients should receive an appropriate type and dosage of antibiotic prophylaxis. Surgeons must scrub with a suitable anti-microbial soap or alcohol-based hand rub before entering the operating theater. All efforts should be made to maintain normo-thermia, hyper-oxygenation, and normal glucose levels during surgery. Hospitals should use the surgical safety checklist to improve quality control measures for the prevention of SSI.
